# Comparison of distance measures in spatial analytical modeling for health service planning

**DOI:** 10.1186/1472-6963-9-200

**Published:** 2009-11-06

**Authors:** Rizwan Shahid, Stefania Bertazzon, Merril L Knudtson, William A Ghali

**Affiliations:** 1 Department of Geography, University of Calgary, 2500 University Drive NW, T2N 1N4, Calgary, AB, Canada; 2 Department of Medicine and Community Health Sciences, University of Calgary, 3330 Hospital Drive NW, T2N 1N4, Calgary, AB, Canada

## Abstract

**Background:**

Several methodological approaches have been used to estimate distance in health service research. In this study, focusing on cardiac catheterization services, Euclidean, Manhattan, and the less widely known Minkowski distance metrics are used to estimate distances from patient residence to hospital. Distance metrics typically produce less accurate estimates than actual measurements, but each metric provides a single model of travel over a given network. Therefore, distance metrics, unlike actual measurements, can be directly used in spatial analytical modeling. Euclidean distance is most often used, but unlikely the most appropriate metric. Minkowski distance is a more promising method. Distances estimated with each metric are contrasted with road distance and travel time measurements, and an optimized Minkowski distance is implemented in spatial analytical modeling.

**Methods:**

Road distance and travel time are calculated from the postal code of residence of each patient undergoing cardiac catheterization to the pertinent hospital. The Minkowski metric is optimized, to approximate travel time and road distance, respectively. Distance estimates and distance measurements are then compared using descriptive statistics and visual mapping methods. The optimized Minkowski metric is implemented, via the spatial weight matrix, in a spatial regression model identifying socio-economic factors significantly associated with cardiac catheterization.

**Results:**

The Minkowski coefficient that best approximates road distance is 1.54; 1.31 best approximates travel time. The latter is also a good predictor of road distance, thus providing the best single model of travel from patient's residence to hospital. The Euclidean metric and the optimal Minkowski metric are alternatively implemented in the regression model, and the results compared. The Minkowski method produces more reliable results than the traditional Euclidean metric.

**Conclusion:**

Road distance and travel time measurements are the most accurate estimates, but cannot be directly implemented in spatial analytical modeling. Euclidean distance tends to underestimate road distance and travel time; Manhattan distance tends to overestimate both. The optimized Minkowski distance partially overcomes their shortcomings; it provides a single model of travel over the network. The method is flexible, suitable for analytical modeling, and more accurate than the traditional metrics; its use ultimately increases the reliability of spatial analytical models.

## Background

Health service research is concerned with the investigation of how social, financial, organizational, technological, and behavioral factors affect access to health care, the quality and cost of health care, and ultimately health and well-being [1]. Distance plays a vital role in studies assessing spatial disease patterns as well as access to hospital services. In a highly complex health care environment, even micro-geographic differences in the availability of tertiary services can affect access to care [2,3]. The study of distances from patient homes to the nearest hospital is an example where distance is often studied as a crude but objective indicator of geographic accessibility to hospital services [4]. In such situations, the measurement of actual travel distance (or travel time) on a road network is clearly the most appropriate method [5]. Health service research, however, encompasses a much broader investigation area, where spatial analytical models are employed to assist in the provision of effective accessibility to health care services. Distance is often used indirectly in these types of analysis as one of the parameters defining the model's thrust and its results.

In a rapidly changing physical and social environment, transportation means and travel modes change quickly as do epidemic transmission modes, overturning traditional ways of conceptualizing and measuring distance [6]. A commonly-used distance metric is the Euclidean distance, a straight line distance measurement between two points, 'as the crow flies' [7,8]. This method is simple and intuitive, but very few are the applications where it can yield accurate distance estimates. An alternative, well-known distance metric is the Manhattan, or taxi-cab distance: as its name suggests, it is most appropriate for grid-like road networks, typical of many North American cities, characterized by a rectangular city block pattern. The Manhattan metric measures distance between points along a rectangular path with right angle turns [9,10]. Most commonly, travel along road networks involves a mixture of Euclidean, Manhattan, and curvilinear trajectories. There is no firm consensus on methods for selecting a distance metric [11], nor is there much published information on the extent to which Euclidean, Manhattan, and road distances relate to one another in applied distance analysis [12,13].

Travel along a complex, or mixed network can be usefully modeled by a class of distance metrics, known as Minkowski distance [14], which is a general distance metric, of which the Euclidean and Manhattan metrics are special cases. This array of metrics provides flexibility and generality, in that, within a single class of metrics, a range of parameters can be selected; therefore, a single yet flexible method for measuring distance can be defined for the optimal estimation of distance on a variety of empirical road networks. One further important aspect is the fundamental role of time in accessing health care services: if distance is a crude estimate of accessibility, travel time is a more relevant estimate. Travel time computation is no longer a prohibitively time consuming and computationally intensive task, thanks to powerful GIS software, hardware, and rich road network datasets [14-17]. However, actual travel time on a road network is highly variable due to local (spatial and temporal) conditions which are hardly predictable and controllable. Because of this characteristic, travel time computations lack general validity, requiring adjustments to account for specific temporal conditions, e.g., weekend vs. weekdays, rush vs. non-rush hours, season, and weather, as well as local spatial conditions, e.g., local traffic congestion, lane closures, or proximity to amenities or popular destinations. All these reasons hamper the implementation of travel time computations in spatial analytical models, since even local analytical models require the definition of a single rule for distance measurement. A crude solution to this problem is the use of average travel time in spatial models; a more realistic solution can be obtained through the use of Minkowski distance: an optimal value of Minkowski distance can be selected to model travel time on a complex road network.

Spatial data tend to exhibit characteristics that negatively impact the statistical properties of quantitative models, decreasing their reliability: spatial analytical models are designed to mitigate these negative effects. The most crucial properties of spatial data are spatial dependence (near things tend to be more similar than distant things) and non-stationarity (inconstant variability of phenomena across space) [18]. Two broad categories of spatial analytical models include spatially autoregressive (SAR) methods, which deal with spatial dependence [19], and geographically weighted (GWR) methods, which deal with spatial non-stationarity [20].

In empirical situations, spatial dependencies and non-stationarities take up specific forms, which are a function of many factors, including the nature of the phenomena under investigation and the representation of space underpinning the model. For this reason, a simplistic application of spatial analysis, one that does not carefully model the salient aspects of phenomena, often fails to fulfill the model's primary objective, which is to enhance the model reliability. The transition from a simplistic to a customized implementation of spatial analysis requires the calibration of each parameter defining the analysis: one of the most crucial parameters, affecting the analytical results, is the distance measurement method.

Cardiac catheterization is a procedure that is performed to determine presence or absence of coronary artery blockages. The procedure involves the percutaneous insertion of a catheter into the arterial system, after which it is guided into the aorta where the coronary arteries are positioned. Contrast dye is then injected into the coronary arteries so that blockages can be located and identified. In some instances, cardiac catheterization can lead to immediate use of percutaneous coronary intervention with balloon angioplasty and the insertion of coronary stents that open up partially or completely blocked arteries to restore blood flow. In some instances, this procedure is performed in stable patients where distance and travel times are a minor concern. In other instances, however, the procedure is done urgently, and for such situations, consideration of distances and travel times become a central consideration in the planning of health services.

In the context of an applied study of distance between patient residence and a tertiary cardiac catheterization facility in a large city, this paper analyzes the effectiveness of a selection of distance metrics in providing a useful model of travel distance and travel time along an urban road network. The comparison of different metrics leads to the identification of a metric that is conceptually sound and computationally effective. The metric thus identified is experimentally used in a spatial autoregressive model analyzing the spatial distribution of cardiac catheterization cases in the city.

## Methods

### Study Area

The study area encompasses the City of Calgary, one of the largest Canadian cities, with approximately 1 million residents [21], distributed over a large geographic area (roughly 750 Km^2^), characterized by diversity of population, housing type, residential density, and accessibility to heath services.

Cardiac catheterization is an invasive procedure for patients experiencing cardiovascular symptoms and defines coronary anatomy, left ventricular and valvular function; it provides important prognostic information for individuals affected by cardiovascular conditions [22]. During the study period the procedure was only performed at the Foothills Medical Centre, located in the northwest of the city.

### Data Sources

Three types of data are used in this study: cardiac catheterization patient database, postal code locations, and the Calgary road network. Cardiac catheterization patient data were obtained from the Alberta Provincial Project for Outcome Assessment in Coronary Heart Disease (APPROACH), an ongoing data collection initiative, begun in 1995, producing information on all patients undergoing catheterization in Alberta [22]. The data are released at the postal code spatial aggregation level. Data were extracted for Calgary residents only and catheterizations performed over the year 2002, resulting in a total of 2, 445 catheterization cases, distributed over 2, 138 postal codes.

A postal code conversion file (PCCF) [23] was obtained from Statistics Canada. Only postal codes that have at least one catheterization case are retained for the analysis. It shall be observed that postal code locations refer to the primary residence of catheterization patients, not to the place where symptoms were felt or where emergency care was first administered.

The Calgary road network data were obtained from the University of Calgary data holdings, based on street information collected and compiled in 2005 by DMTI Spatial [24]. This road network was used to calculate shortest road distances from patient residence location to hospital for cardiac catheterization services.

### Distance Metric Calculation

Straight line (Euclidean) distance and Manhattan distance are often used in health service research [25]. Each of these distance metrics may appropriately estimate distance in some parts of a study area, but their application at the city level tends to yield large errors in areas that depart from the dominant pattern, and may lead to highly inaccurate distance estimations. One of the reasons for using Euclidean and/or Manhattan distance is the relative ease of their implementation; in contrast, it is more problematic to design algorithms implementing actual road network distance in spatial analytical models.

In order to reduce the error associated with the Euclidean and Manhattan metrics while maintaining the computational simplicity of a single, intuitive mathematical formula, the general Minkowski metric is examined, to devise a single method that best approximates the average pattern of an empirical road network. Optimizing values of the Minkowski formula are calculated for road distance as well as travel time; the results are compared with more traditional distance measures in the context of assessing geographic accessibility to cardiac facilities. The Minkowski distance has the potential to provide a more accurate estimate of road network distance and travel time than the Euclidean and Manhattan metrics.

A set of 2, 138 distances between each patient's postal code of residence and the Foothills hospital are calculated according to each of the distance measurement methods considered. The geographic locations of each postal code from the PCCF and the hospital are recorded in latitude and longitude; therefore, in order to implement distance computations, the road network is projected using an equidistant projection system, which is chosen in order to preserve distance and produce consistent distance measurements [26]. Latitude and longitude coordinates are then converted into Eastings and Northings, i.e., *x *and *y *values, expressed in kilometers. Alternative methods could have been used, for example the great circle distance formula [27], which, however, provides rougher estimations. The ArcGIS 9.3 [28] Geometry calculator was used to calculate the *x *and *y *coordinates based on the projected dataset and the resulting *x *and *y *values were used in the distance formulas defined below.

Euclidean [7,8], Manhattan [9,10], and Minkowski [14] distance can be calculated by the formula:(1)

where, for this application:

*d *is the distance between a patient's residence and the hospital;

*x*_*i*_, *y*_*i *_are the geographic coordinates of the centroid of each postal code of residence;

*x*_*j*_, *y*_*j *_are the geographic coordinates of the Foothills hospital.

**Figure 1:**
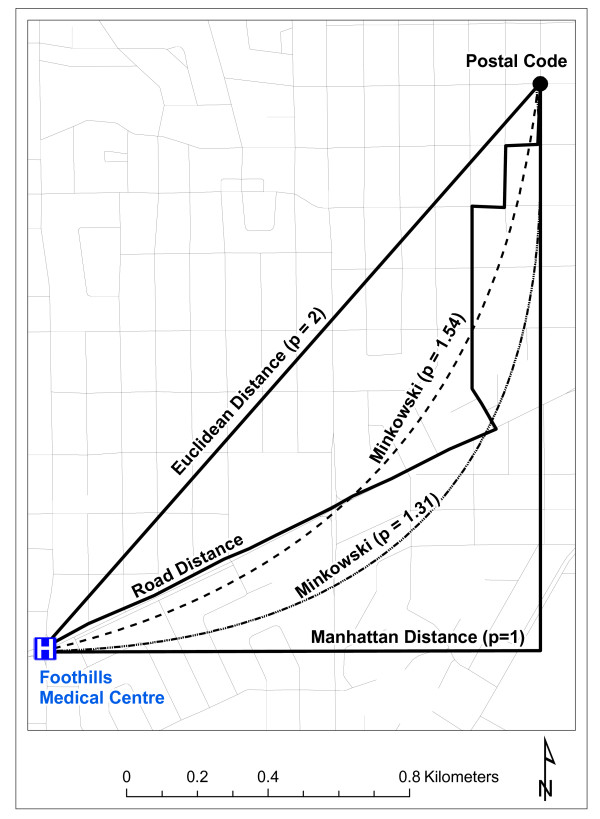
**Visual illustration of road distance and distance metrics**.

The generic *p *parameter in Equation 1 can be replaced by the value 2 to yield the well known Euclidean distance; the value 1 would yield the Manhattan distance, and all the intermediate values in the in the [1 <*p *< 2] interval yield an array of Minkowski distances (Figure 1).

### Road Network Distance and Travel Time Calculation

The computation of road network distances (shortest distance between two locations along a road network) is implemented directly in GIS software [28] using a shortest path algorithm. It is recognized *a priori *that this method depicts the actual travel trajectory and is likely to produce the most accurate distance estimates for patient travel routes to a hospital. Likewise, travel time calculated over the road network using an appropriate algorithm is likely to yield the most accurate travel time estimates. The basic need for road distance calculation is a road network with information on all the segments constituting the network, as well as on all existing constraints such as prohibited left or right turns, one way streets, etc.

Travel time calculations, likewise, are implemented in GIS software [28]. Additional information required for travel time calculation include estimated speed and length of road segments, along with an algorithm capable of taking all these factors into consideration [15]. The Network Analyst extension within ArcGIS [28] enables the modeling of spatial networks and provides the tools for road distance calculations [28,29] and travel time calculations [15,16] from multiple postal code locations to a hospital. Figure 1 illustrates road network distance, along with the distance metrics considered in this study.

### Computing Minkowski Coefficients

A simple procedure can be implemented to select, within the [1 ≤ *p *≤ 2] interval, the value of the parameter *p *in the distance formula that best approximates distance along a given road network. In light of the applied focus of this analysis, an empirical solution to this problem is sought for the set of 2, 138 patient-to-hospital distances. As a first step for determining the best Minkowski distance, Equation 1 is transformed into Equation 2:(2)

Two new quantities are defined as *X *= (*x*_*i *_- *x*_*j*_) and *Y *= (*y *_*i*_- *y*_*j*_), and replaced in Equation 2, which is also further modified by means of a logarithmic transformation. Equation 2 is solved for *p *values in the [1 ≤ *p *≤ 2] interval, sampled at regular intervals. The set of distances thus calculated are considered approximations of the road distance. A simple regression model is defined, where the dependent variable is the road distance, and the independent variable is, in turn, the distance obtained by each *p *value. Goodness-of-fit, residuals, and other regression diagnostics are then compared over the entire interval [30,31]. This simple method helps assess and rank the various coefficients, identifying the one that produces the highest *R*^2 ^value, which is considered the best Minkowski coefficient.

The procedure is then modified to determine the optimal Minkowski coefficient for travel time: conceptually, this experiment is less straightforward, because travel time is a measure of time, whereas Minkowski remains a measurement of distance in space. Once travel time is computed, in order to make the transition between time and space, in terms that are valid both conceptually and computationally, the concept of speed, a simple ratio between space and time, is introduced. Average speed over the city is calculated, yielding the following values: the average distance traveled in one hour is 58.71 km; conversely, the average time required to travel 1 kilometer is 1.02 minutes. For the sake of simplicity, in order to make the argument more intuitive, these values were rounded to an average speed of 60 km/h or 1 minute to travel 1 kilometer. It shall be observed that these values are obtained under optimal conditions, i.e., without considering delays due to rush hour, traffic congestion, traffic lights, stop signs, road closures, weather, etc.

Travel time calculations are then used as the dependent variable, while the independent variables remain unchanged. To this end, actual travel times are converted to distances, using the average travel time, and replaced in the procedure for the calculation of the optimal Minkowski *p *value to approximate travel time. Once the two optimal Minkowski coefficients are identified, standard descriptive statistics are used to analyze and compare the four distance metrics and the two empirical distance measurements.

## Results

**Figure 2:**
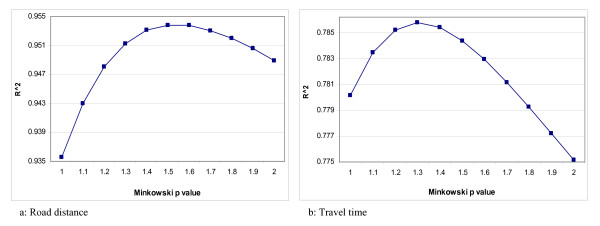
**Determination of optimal *p *values for Minkowski distance**.

The results of the regression models estimated to identify the optimal Minkowski coefficients are summarized in Figure 2. Figure 2a summarizes the regressions for the road distance coefficient optimization, and Figure 2b for the optimization of the travel time coefficient. Figure 2 shows different values of a goodness-of-fit indicator (*R*^2^) obtained from a regular sample of *p *values throughout the [1 ≤ *p *≤ 2] interval. Figure 2a shows that the *R*^2 ^increases gradually for *p *values between 1.00 and 1.50, it levels out for *p *values between 1.50 and 1.60, and then decreases again for *p *values ranging from 1.61 to 2.00. The range of values for a 'best' Minkowski *p *value is therefore in the range of 1.50 ≤ *p *≤ 1.59. The overall pattern displayed by *R*^2 ^is very important, as it indicates a consistent behavior over the interval. In practice, this plot represents *R*^2 ^as a function of the Minkowski coefficient. The observed trend suggests that indeed the function does have a unique maximum in the range 1.50 ≤ *p *≤ 1.59. It is possible, therefore, to confidently accept a value within this range as the maximum. Less consistent trends would decrease the confidence in the choice of an optimal value. For simplicity sake, one single value, *p *= 1.54, is chosen as the optimal Minkowski coefficient for the road distance.

Based on this result, Equation 1 can now be re-written as:(3)

Equation 3 expresses the Minkowski distance metric that best approximates distance on the road network. This metric is consequently used to produce a model of the distance separating cardiac patients from the Foothills hospital. It is worth noting the coefficient identified is approximately halfway between the Euclidean (*p *= 2) and Manhattan (*p *= 1) distance, leading to two alternative interpretations: either the dominant pattern of Calgary road network is an intermediate trajectory between the Euclidean and Manhattan models, or the road network, overall, is a mixture of the two patterns, with approximately equal contributions from each one.

Figure 2b shows the corresponding *R*^2 ^values for the travel time optimization of *p *in the [1 <*p *< 2] interval. The *R*^2 ^increases gradually for *p *values between 1.00 and 1.25, then it levels out for *p *values between 1.25 and 1.35. The curve decreases more sharply from 1.35 to 2.0. The range of values for the best candidate of *p *value for travel time is in the range of 1.25 ≤ *p *≤ 1.35. The values of *R*^2 ^suggest the best *p *value in this range, which is 1.31. Based on this result, the general Minkowski formula (Equation 1) can be written as(4)

where *tt *is the travel time that is based on the average speed, whereby one minute corresponds to one kilometer. The *p *value for travel time (1.31) is lower than the *p *value for distance (1.54), suggesting that travel pattern is closer to the Manhattan model if travel time is considered.

Table 1 presents summary descriptive statistics of the five measured distances. Road network distances range from 0.43 to 30.68, with a mean of 11.82 km distance from a patient's residence to the Foothills Medical Centre. Of all the methods used to measure distance, it displays the second largest standard deviation and range. Travel time can be immediately compared with all the distance metrics, as 1 minute corresponds to 1 kilometer: in comparison with road network distance, travel time displays a lower standard deviation and a shorter range, with a minimum travel time of 1.51 minutes, and a maximum of 26.76 minutes, under optimal conditions. Conversely, its mean displays the highest value, 12.11 minutes. Shorter range and higher mean are likely due to the opposite effects of speed limits on road segments of different length: in general, longer travel paths include large segments that occur on major roads, where speed limits are higher, whereas shorter trips tend to occur on minor roads, which are associated with lower speed limits. As a consequence, a model that takes speed into consideration produces a shorter travel range. Consistently, the standard deviation is lower. The higher mean value suggests that overall speed limits tend to slow down travel; that is, segments with low speed limits have a large impact on the overall travel pattern throughout the road network.

**Table 1:** Summary descriptive statistics of distance measurements and metrics

Distance Metric	Min.	Max.	Range	Mean	Std.Dev.
Road Distance	0.43	30.68	30.25	11.82	5.27
Travel Time (*)	1.51	26.76	25.26	12.11	4.92
Euclidean Distance	0.40	25.45	25.05	9.37	4.63
Manhattan Distance	0.49	35.69	35.20	11.78	5.91
Minkowski Dist. (p = 1.54)	0.41	28.12	27.71	9.93	4.91
Minkowski Dist. (p = 1.31)	0.43	30.37	29.94	10.45	5.18

(*) Expressed in Km./minutes, where 1 Km. = 1 minute

Euclidean distance tends to underestimate road distance, as shown by the mean and range; the standard deviation is lower than for road distance, with values that are fairly close to those for travel time. This is probably due to the smoothing effect of a uniform distance model, which produces lower values than actual road distance for curvilinear segments and the most complex paths.

Manhattan distance tends to overestimate road distance and produces values consistently larger than those of Euclidean distance. Its mean value is very close to the road distance mean, but it also presents the largest standard deviation and the largest range. This may suggest that the Calgary road network contains several parts that follow the Manhattan pattern (hence a similar mean value), but the presence of different patterns in the same network increases its error (large standard deviation).

In comparing Euclidean and Manhattan distances, Manhattan distance produces a close approximation of the mean, and only slightly overestimates the standard deviation, whereas Euclidean largely underestimates both values. For the range, both metrics produce approximately the same error, though with opposite sign. This suggests that, overall, Manhattan is a better model than Euclidean for the Calgary road network.

The value of *p *= 1.54 best approximates road distance in the Minkowski formula. Indeed minimum and maximum values are close approximations, whereas mean and standard deviation underestimate road distance. This result can be considered satisfactory, as it indicates that the error is minimized for individual measurements, but overall the method displays the aforementioned smoothing effect, whereby mean measurements tend to be slightly smaller than actual ones, with overall lower variations around the mean. It shall be observed that this distance metric is approximately half-way between Euclidean and Manhattan; however, all the descriptive statistics present values that are closer to the Euclidean than the Manhattan results.

The value of *p *= 1.31 best approximates travel time in the Minkowski formula. Interestingly, the descriptive statistics suggest that this metric is the best approximation of road distance; indeed a close approximation. As noted, travel time presents features that differ from distance, i.e., larger mean and lower range: this combination is hard to achieve by the class of metrics considered, because *p *values closer to 1 (Manhattan) produce larger means, whereas *p *values closer to 2 (Euclidean) produce lower ranges. In light of these considerations, a relatively low *p *value, such as *p *= 1.31 most closely approximates this pattern, rendering a model that is less extreme than Manhattan, as shown by a lower standard deviation. Overall the metric *p *= 1.31 provides a model of travel that is intermediate between measured road distance and travel time measured under optimal conditions. This is supported by almost all the descriptive statistics; the greatest shortcoming of this metric is its poor approximation of the mean. This value produces the best of all the metrics examined.

Finally, it shall be noted that the travel time pattern would be very different if measurements were to consider less favorable conditions. The most common impediments to fast travel in Calgary include traffic congestion, e.g., rush hour, and severe winter weather conditions. Traffic congestion tends to affect major roads more heavily, where feasible speed can easily be reduced by 20-25% of the speed limit, whereas its effect is generally lesser on minor roads. Severe winter weather conditions are likely to have a comparable effect on major roads, but they will also have comparable or worse effects on minor roads. However, a speed reduction from 80 to 60 km/h on long road segments significantly impacts travel time, lowering the maximum and range values, and increasing the mean value. Conversely, an equivalent or greater speed reduction from 50 to 40 or 35 km/h on shorter road segments is likely to have only a minor impact on the overall travel time. Seeking to approximate such travel pattern via a Minkowski *p *value is therefore unlikely to produce better results.

### Differences between distance measurements and distance metrics

Table 2 presents a selection of summary statistics of the differences between each empirical measurement and the distance metrics considered. These are only global results, overshadowing the performance of each distance model throughout the city. These results suggest that, globally, the differences produced by each model are modest.

**Table 2:** Summary of differences between distance measurements and metrics

Difference	Min.	Max.	Range	Mean	Std.Dev.
Road -- Euclidean	0.01	8.11	8.10	2.45	1.29
Road -- Manhattan	0.00	7.64	7.64	1.15	1.04
Road -- Minkowski (p = 1.54)	0.01	8.02	8.01	1.89	1.15
Road -- Minkowski (p = 1.31)	0.00	7.92	7.92	1.37	1.15
					
TT(*) -- Euclidean	0.01	15.25	15.24	2.95	1.95
TT(*) -- Manhattan	0.00	23.49	23.49	2.02	1.83
TT(*) -- Minkowski (p = 1.54)	0.00	17.39	17.39	2.51	1.87
TT(*) -- Minkowski (p = 1.31)	0.00	19.21	19.20	2.20	1.81

(*) Expressed in Km./minutes, where 1 Km. = 1 minute

**Figure 3:**
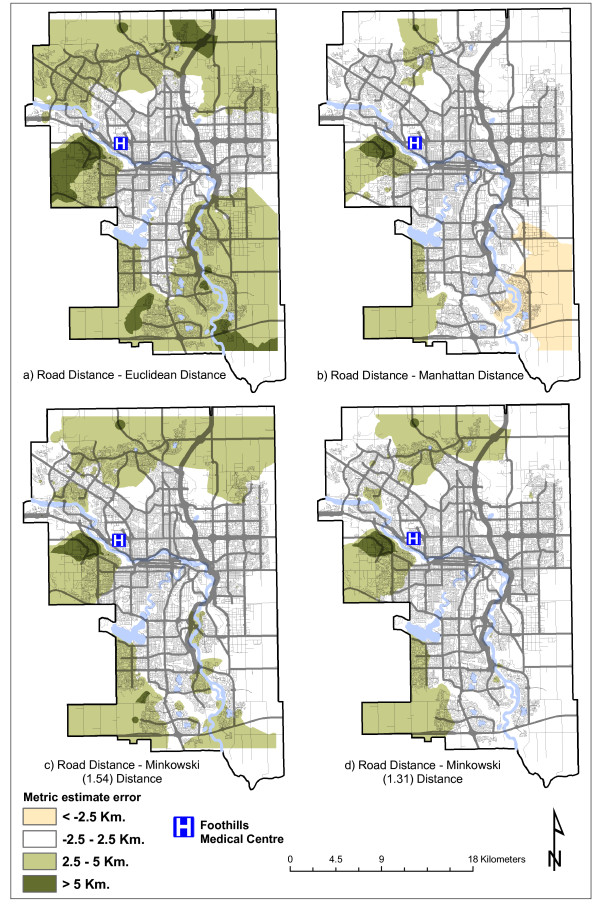
**Differences between road distance and distance metrics**.

**Figure 4:**
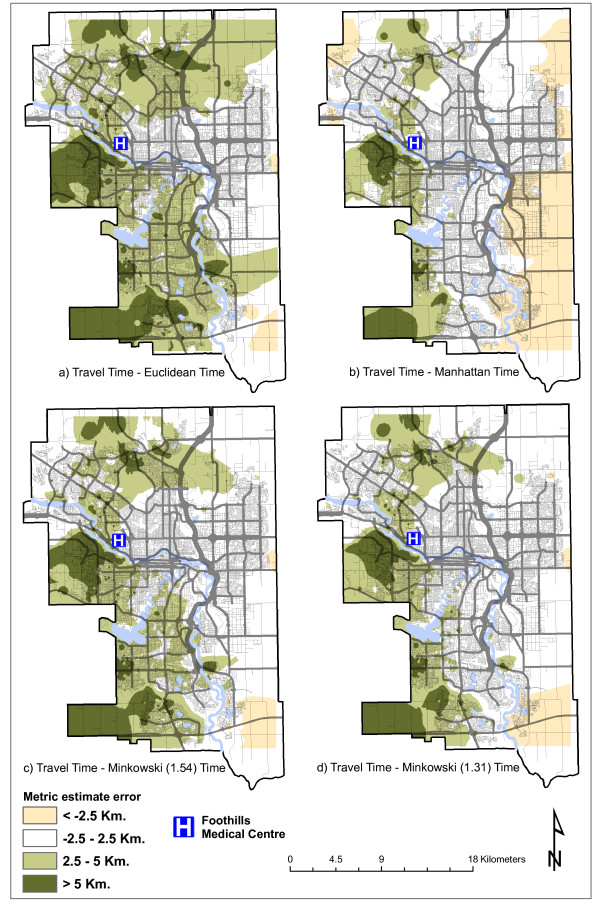
**Differences between travel time and distance metrics**.

For road distance, the smallest differences are achieved by the Manhattan and Minkowski (*p *= 1.31) metrics, with Manhattan producing the overall best result. The same metrics produce the best results for travel time, with Minkowski (*p *= 1.31) producing the lowest standard deviation. Median and mean differences are lower than 2 kilometers and just over 2 minutes, respectively. However, standard deviations tend to be quite high, relative to the mean. Figure 3 and Figure 4 illustrate graphically these differences, providing greater spatial detail. Differences between road distance and each distance metric are presented in Figure 3, and those between travel time and each metric in Figure 4. The figures display spatially the magnitude of the error associated with each distance metric in each part of the city.

With respect to road distance (Figure 3), Euclidean distance produces only negative errors, but in some peripheral areas of the city these errors are large in absolute value. Manhattan distance produces mostly positive errors, and overall it produces better results, as the areas characterized by the highest absolute errors are reduced to a triangle west of the hospital and the far southeast corner of the city. The Minkowski metric with *p *= 1.54 improves over the Manhattan metric results, by resolving the area of large residuals in the southeast corner; conversely, the area of high residuals west of the hospital is moderately larger. The best results are produced by the Minkowski metric with *p *= 1.31, as for the entire eastern part of the city errors are contained in the interval 0-2.5 km, and greater errors remain only in some peripheral northwest areas.

The area west of the hospital consistently emerges as an outlier, despite its close distance to the hospital. Careful observation of the topography of the area reveals that the hospital is located on the east side of a hill, with a river running to the west of the hill. Therefore, no immediate access is possible from the west side of the river to the hospital, and patients are left with no option but to travel a considerable distance either northbound or southbound to access the nearest bridge. The presence of the hill further amplifies this distortion.

With respect to travel time (Figure 4) the improvement obtained by the two Minkowski metrics is more evident throughout the city. Areas of large residuals remain in the far southeast and southwest corners, and, in general each metric performs worse in the west than in the east part of the city. The latter observation is counterintuitive, since the hospital is located in the northwest; however, that part of the city, closer to the foothills, is characterized by a more complex topography. Moreover, in a city like Calgary, urban design and age of communities play an important role, as, over the decades, rectangular patterns have alternated with such patterns as crescent and cul-de-sac, and other typologies of urban connectivity. Likewise, it shall be observed that the most peripheral areas are very recent developments, and likely the planned road connections had not been completed during the study period.

### Spatial analytical modeling application

**Figure 5:**
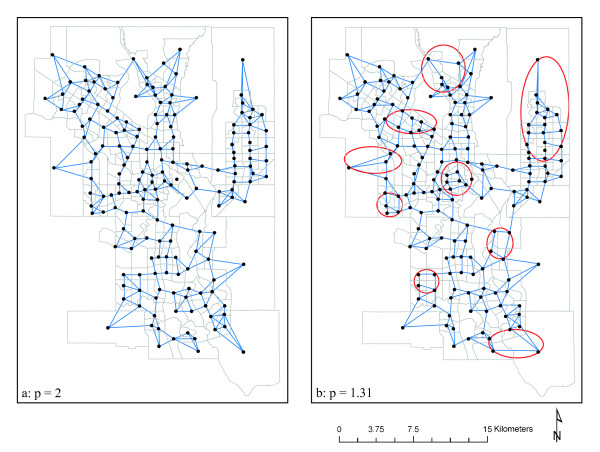
**Neighborhood configurations determined by different distance metrics**.

Within the scope of a larger project, the association between cardiovascular disease and socio-economic variables was recently analyzed [32]. Specifically, the relationship between cardiac catheterization and socio-economic variables was analyzed by means of a multivariate spatially autoregressive model. While distance does not explicitly enter in these models, it is one of the key parameters defining the spatial weight matrix (19), which represents the neighborhood definition, hence the model's ability to cope with spatial dependencies, and ultimately the reliability of the model estimates. Figure 5 provides an example of how different distance metrics affect the neighborhood configuration defined by a spatial weight matrix. Locations in the figure represent census tract centroids, as these relatively larger spatial units are used in these regression models. The lines connecting these locations indicate whether or not 2 close locations are considered neighbors and included in the estimation of spatial dependence. Other parameters contribute to the neighborhood definition: generally the most influential parameter is the number of nearest neighbors, complemented by a distance decay function and a weight [33]. Figure 5a shows the connectivity defined by the Euclidean metric, while Figure 5b corresponds to the Minkowski metric optimizing travel time (*p *= 1.31). A close comparison of the two plots (aided by the superimposed circles) reveals how the modification of the distance metric substantially alters the neighborhood configuration.

Each neighborhood configuration, such as the ones presented in Figure 5, forms the basis for the definition of a spatial weight matrix, which is one of the crucial elements that define a spatial regression model, differentiating it from a standard (non-spatial) model. Through the spatial weight matrix, the neighborhood configuration ultimately affects the variance of the model estimates, hence their reliability. As an example, Table 3 shows the variation in the parameter estimates and the regression diagnostics determined by the two alternative neighborhood configurations depicted in Figure 5. The regression model analyzes the socio-economic variables significantly associated with cardiac catheterization: the main predictors are family status, income, and educational attainments; the spatial distribution of these variables is used to identify areas of social and economic concern. This model thus provides an effective analytical tool to support policy decisions, providing guidance for the initiation of targeted, localized preventative health measures [32].

**Table 3:** Spatial regression analysis for varying neighborhood configurations

		Minkowski *p *value	
		*p *= 2	*p *= 1.31
***Model Variables***			
**Families with children**	β coefficient	-1.88	-1.89
	t test	-7.01	-7.11
**Non-university education**	β coefficient	-2.45	-2.37
	t test	-5.60	-5.47
**Family median income**	β coefficient	1.06	1.10
	t test	4.27	4.45
**Secondary education**	β coefficient	1.30	1.31
	t test	3.63	3.64
***Regression diagnostics***			
**Pseudo-R^2**		0.32	0.32
**Autoregressive Parameter**		0.94	0.98
**Residual Spatial Autocorrelation**		-0.03	-0.02

A succinct summary of the parameters associated with each independent variable is presented in Table 3, along with a small selection of regression diagnostics. While the coefficients (beta) linking each independent to the dependent variable remain substantially unchanged, the Minkowski distance leads to increased values of their associated *t *test: the *t *values increase thanks to a reduction of the variance associated with the estimates. All the independent variables benefit, in varying degrees, from the modified distance model. Likewise, the regression diagnostics indicate that the distance model does not appreciably affect the overall goodness of fit (represented by the pseudo-*R*^2^), but it does noticeably impact the autoregressive coefficient and, more importantly, the spatial dependence in the regression residuals.

These results confirm the importance of an accurate distance model to enhance the reliability of spatial analysis for health service research.

## Discussion

This study compares four different distance metrics, i.e., Euclidean, Manhattan, and Minkowski distance (the latter for two different coefficients), and contrasts them with road distance and travel time, respectively, in the context of applied health services research. The Euclidean metric is the most common and intuitive measure of distance; Manhattan is another common distance measure, but very rarely does either of them provide a close approximation of an empirical road network, such as an urban network. Road distance, conversely, provides an accurate measurement, but it is prone to local features, and does not provide a single model of travel throughout the urban network. Travel time is, arguably, the most relevant estimate of distance, but its calculation introduces further specificities, as temporal anomalies are added to the local features, further reducing its generality. For these reasons, Minkowski distance is a promising solution: it provides a general model of travel throughout an empirical network; it possesses a large range of parameters, which enhance its flexibility; it can be easily calculated; and it can provide a less crude approximation of travel along a road network.

Euclidean distance is widely used in distance analyses in the literature [25] but it tends to underestimate road distance and travel time. Manhattan distance, on the contrary, tends to overestimate road distance and travel time. The use of either of these two metrics in any spatial analysis may result in inaccurate results [10]. Ideally, travel time provides the most accurate estimate of a patient's travel from their place of residence to a given health facility [15]. While accessibility studies may profitably employ this method, its use in spatial analytical models is inhibited by the particularity of its calculation, which tends to be heavily affected by local features, both in space and in time.

A simple, empirical optimization procedure led to the identification of the coefficients, in the Minkowski formula, that best approximate road distance and travel time, respectively. Summary statistics and cartographic representations consistently indicate the value *p *= 1.31 as the best coefficient, i.e., the one that leads to the most accurate approximation of both road distance and travel time. The model of distance based on this coefficient was experimentally introduced in spatial analytical routines, to define neighborhood connectivity and determine the spatial weight matrix for multivariate spatial regression analysis.

The enhanced reliability of the spatial analytical model based on the optimal distance model far outweighs the cost of the computational procedure that leads to the coefficient selection. The advantage of the procedure discussed in this paper can be best appreciated by considering that empirical measurements of distance and travel time cannot be implemented in spatial analytical modeling, exactly because of their empirical nature and the great impact they receive from local features and conditions. For this reason, the optimized Minkowski coefficient represents a valuable compromise between an approach that is often simplistic (i.e., Euclidean and Manhattan metrics) and the ideal, but impractical sophistication of empirical measurements (i.e., road distance and travel time, respectively).

This study has some limitations. Most importantly, it is limited to one class of distance metrics, i.e., Minkowski, whereas other metrics could be considered: Mahalanobis distance is just one example [34]. There are locational inaccuracies in the patient data [35] as well as in the road network; additional errors are likely to derive from the algorithm used for the distance calculations. Travel time calculations are based on assumptions, referred to as optimal conditions that tend to represent an ideal, but unlikely situation. The hypothesis of optimal conditions should be lifted, and more realistic conditions should be entered in the model, e.g., rush hour, or severe winter weather, and should be considered not just individually, but also jointly. An array of optimal Minkowski coefficients should consequently be calculated for the varying conditions, leading to the final identification of a stochastic optimum. The entire procedure is also based on the strong assumption of a single transportation mode: it can be argued that optimal conditions approximate ambulance travel, but the catheterization registry used in this analysis was not limited to patients who were transported directly from their residence to the tertiary catheterization facility. The APPROACH registry also includes patients initially admitted to hospitals or emergency facilities without catheterization facilities who were subsequently transferred to a tertiary center for this procedure under less urgent circumstances. Accuracy in travel route selection and in travel time estimates are most relevant to patients with more emergent cardiac conditions requiring rapid transportation from their residence to the catheterization facility. This limitation can be addressed by a twofold model, optimizing for ambulance and private vehicle travel. Still, road network travel is a reasonable assumption for urban environments, but is unlikely to be the sole mode of transportation to emergency care from rural and remote locations.

The proposed approach was tested, as an example, on a spatial autoregressive model; however, virtually all spatial analytical techniques involve some distance measurement. The impact of alternative distance measurements was examined in this implementation through an analysis of the spatial weight matrix, but distance is likely to impact other analytical techniques in further, different ways [19,20].

Given its advantages and limitations, Minkowski distance appears to be most usefully implemented in spatial analytical modeling; however, other useful applications can be envisaged, particularly in geographic areas characterized by paucity or unreliability of spatial data, or by high dynamism. Examples include urban or regional road networks of countries with poor spatial digital records, or characterized by high population mobility or varying transportations routes, for example due to seasonal variations. In all such cases, a model of travel in the area, obtained by the method discussed in this paper, can provide rough, but reasonable distance estimates, potentially useful for facility planning, or as initial input for more sophisticated analyses.

## Conclusion

The proposed method provides a single model of travel on an urban road network, via the identification of an optimal coefficient within a class of distance metrics, known as Minkowski metrics. The coefficient can be optimized to approximate different distances, e.g., road distance or travel time, under varying conditions. The resulting distance model can be usefully input in spatial analytical models, providing a method for the estimation of less simplistic spatial analytical models, by means of a more accurate representation of distance. Such models yield more reliable estimates, hence more effective tools for health service planning and management.

## Competing interests

The authors have no conflict of interest to declare and each has met the criteria for authorship (including access to the data and role in writing the manuscript).

## Authors' contributions

All authors read and approved the final manuscript. RS compiled the data, performed the distance analyses and calculations, and drafted manuscript. SB conceived the conceptual method to optimize Minkowski metric; performed the spatial analytical implementation; and thoroughly revised the manuscript providing extensive guidance. MLK provided clinical context and comments on the manuscript. WAG participated in study design, contributed in writing manuscript and provided support and guidance throughout the study.

## Pre-publication history

The pre-publication history for this paper can be accessed here:

http://www.biomedcentral.com/1472-6963/9/200/prepub
